# Evaluating the effectiveness of using personal tailored risk information and taster sessions to increase the uptake of smoking cessation services: study protocol for a randomised controlled trial

**DOI:** 10.1186/1745-6215-13-195

**Published:** 2012-10-18

**Authors:** Hazel Gilbert, Stephen Sutton, Richard Morris, Steve Parrot, Simon Galton, Irwin Nazareth

**Affiliations:** 1 Research Department of Primary Care and Population Health, UCL, London, UK; 2 Institute of Public Health, University of Cambridge, Cambridge, UK; 3 Department of Health Sciences, University of York, Heslington, York, UK; 4 Smokefree Camden (Public Health), NHS Camden, London, UK

**Keywords:** Smoking cessation, Stop smoking clinics, Computer-tailoring, Personalisation, Risk information

## Abstract

**Background:**

Although government-funded specialist smoking cessation services in England offer advice and support to smokers motivated to quit, only a small proportion of smokers make use of this service. Evidence suggests that if smokers are proactively and personally invited to use services, use will be higher than with a standard referral made by health professionals. Computer-based systems generating personalised tailored communications also have the potential to engage with a larger proportion of the smoking population. In this study smokers are proactively invited to use the NHS Stop Smoking Service (SSS), with a personal computer-tailored letter and the offer of a no-commitment introductory session designed to give more information about the service. The primary objective is to assess the relative effectiveness on attendance at the NHS SSS, of proactive recruitment by a brief personal letter, tailored to individual characteristics, and invitation to a taster session, over a standard generic letter advertising the service.

**Method/design:**

This randomised controlled trial will recruit smokers from general practice who are motivated to quit and have not recently attended the NHS SSS. Smokers aged 16 years and over, identified from medical records in participating practices, are sent a brief screening questionnaire and cover letter from their GP. Smokers giving consent are randomised to the Control group to receive a standard generic letter advertising the local service, or to the Intervention group to receive a brief personal, tailored letter with risk information and an invitation to attend a ‘Come and Try it’ taster session. The primary outcome, assessed 6 months after the date of randomisation, is the proportion of people attending the NHS SSS for at least one session. Planned recruitment is to secure 4,500 participants, from 18 regions in England served by an NHS SSS.

**Discussion:**

Personal risk information generated by computer, with the addition of taster sessions, could be widely replicated and delivered cost effectively to a large proportion of the smoking population. The results of this trial will inform the potential of this method to increase referrals to specialised smoking cessation services and prompt more quit attempts.

**Trial registration:**

Current Controlled Trials ISRCTN76561916

## Background

The prevalence of smoking in the UK has fallen from 25% to 22% and 23% to 21% between 2005 and 2008 in men and women, respectively [1]. While government targets to reduce smoking prevalence have been achieved [2], there is no room for complacency. Smoking remains a major cause of ill health and mortality, accounting for approximately 18% of all deaths in adults aged over 35 years in 2007 [3]. Furthermore, national statistics from 2008 figures show a widening gap in smoking prevalence between those in professional and managerial occupations, and routine and manual workers; 29% of the latter still smoke, rising to 42% of those currently not employed [1].

Government-funded specialist smoking cessation services were implemented in 1999 in Health Action Zones, and were rolled out throughout England in 2000 [4]. These services offer intensive advice and support to smokers motivated to quit, in group or one-to-one sessions. However, most smokers will not attend formal cessation programmes, preferring to quit on their own, consequently such programmes are consistently underused [5-8]. In 2007, 74% of current smokers in Great Britain reported that they want to quit, and 31% had made an attempt to quit in the previous year [9]. In 2001 to 2002, 2.01% of the adult smoking population in England set a quit date using the National Health Service (NHS) Stop Smoking Services (SSS) [10], and even now this figure is estimated to be < 5% [11-13]. Thus, despite a desire to quit, only a small proportion use the free service provided by the NHS [14].

A wide range of factors, such as lack of availability and accessibility, perceived inappropriateness of the service, a perception that help is not necessary, or a sense of a lack of empathy from health professionals, as well as a lack of readiness to quit, will bar smokers from seeking help [15,16]. The literature also suggests that many smokers are unaware of, or have insufficient knowledge of or inadequate information about the services available [16,17] and this lack of knowledge can also lead to the belief that ‘it wouldn’t help me anyway’ [16].

Health professionals are guided to offer brief advice and refer smokers to the services, but the percentage of smokers receiving such advice is small, and only 9% were referred to the cessation services in 2007 [3]. Moreover, these smokers are generally expected to follow-up their referral and contact the service themselves to make the appointment. Lichtenstein [7] evaluated an intensive and standardised referral protocol, employing a more proactive method of recruitment and referral by inviting smokers to an intervention with a strong referral message to the service and offering information about what attendance at the service would involve. This intervention included an assessment, measurement of expired-air carbon monoxide (CO) level with an interpretation, a 10-min video of a stop smoking group program featuring former successful group members, a voucher fee waiver, and immediate scheduling of the smoker for the group. With this intervention, 11.3% of smokers attended the first session of the cessation programme, compared with 0.006% of the control group who received brief advice only.

While recruitment methods to cessation services generally employ a reactive approach, in which smokers are expected to seek out and approach the service [16], evidence suggests that if smokers are proactively and personally invited to use the services, the resultant use will be higher than standard referral by health professionals, or open advertising. In a study exploring the acceptability of proactive contact offering cessation services to smokers, 92.8% found it acceptable for the health service to contact people to offer assistance, and 55.7% said they were likely to take up the offer of individual counselling [16]. This could be an overestimation of actual take-up of the service, but suggests that proactive contact is acceptable and that smokers are open to intensive counselling.

In a study by Murray and colleagues [11,18], general practices identified all patients who were recorded as current smokers or with no status recorded, and proactively informed them by letter about the stop smoking services, giving the option of being contacted by an advisor. The proportion of current smokers expressing interest was 13.8%, suggesting that more than the current 5% of the smoking population setting quit dates within the NHS are interested in receiving help. Hence, novel methods of marketing to engage interested smokers are needed in order to encourage use of the services. Furthermore, general practices were randomized to an intervention group or to a control group. Smokers in practices allocated to the intervention group indicating that they would like to speak to an advisor were contacted within 8 weeks by a researcher trained as an advisor and offered advice and an appointment. Smokers in control group practices received no further contact. Murray reported a 7.7% increase in smokers using the NHS SSS in the intervention group over the control group at the 6-month follow-up, and an increase of 1.8% in validated abstinence in those smokers requesting contact, over the control group (4% *vs.* 2.2%).

The study by Murray and colleagues [18] was the first to assess a proactive method of recruitment to attract smokers into the services. However more personal methods of recruitment such as the use of tailored self-help materials, intended to meet the needs of one specific person, based on characteristics unique to that person [19] could further enhance recruitment. The development of these materials has enabled the generation of highly-tailored behavioural feedback reports for smoking cessation [20]. A computer-based system developed by two of the investigators (HG and SS) to generate individually-tailored feedback reports designed to encourage and help smokers to quit demonstrated a positive effect when used as an adjunct to telephone counselling (via the national Quitline) [21], a finding consistent with other studies [22]. These computer-based systems offer a method for further personalising communications to patients and have the potential to engage with and recruit a larger proportion of the smoking population.

In this study we extend the work of Murray and colleagues [18] by providing a more intensive intervention using computer-tailored feedback to deliver personalised risk information to invite and encourage people to attend the NHS SSS. In addition smokers are offered a no-commitment taster session designed to inform them about the service and what it offers.

Intensive clinical treatment is particularly important for: (1) smokers at high risk because of chronic conditions; and (2) heavily dependent smokers who have been unsuccessful in previous attempts [5]. Furthermore, a long-term aim of the NHS SSS is to help disadvantaged people to stop smoking. However, government targets to reduce smoking prevalence in the UK in manual groups to 26% by 2010 have not been reached [23]. Thus, as part of this strategy the delivery of cessation services to poorer communities has been a priority [10]. While the services have succeeded in attracting smokers from disadvantaged areas [10,15,24], unacceptable smoking-related health inequalities persist [1]. An advantage of the proactive recruitment method is the ability to target at risk groups. Smokers from the most disadvantaged areas are more interested in receiving help than smokers from areas of low deprivation [11], thus more attractive methods to inform and engage this group are needed, including the use of medical information on chronic illnesses and high dependence to tailor our communications with smokers.

It is possible that by using methods of direct mail contact, smokers not ready or motivated to quit may be encouraged to attend the SSS, but would be less likely to quit than self-referred patients [14]. Traditionally smokers with an intention to quit in the next 2 weeks are targeted in the NHS for attendance at specialist clinics, but planning to quit in the near future should not be taken as the only indicator of interest in quitting. Studies have shown that smokers stating that they have no plans to quit have taken part in cessation programmes [25], and evidence from a recent trial suggests that proactive recruitment can successfully engage smokers with no immediate plans to quit in quitting activity [26]. There is also evidence of smokers quitting without entering a preparation stage or planning to quit [27]. Thus, in this study, we include those whose intention to quit is more distant, and those who express an interest in receiving help to quit.

The primary objective of the study is to assess the relative effectiveness on attendance at the NHS services of at least one session, of proactive recruitment by a brief personal letter, tailored to individual characteristics available in medical records, and invitation to a taster session to provide information about the NHS services, over a standard generic letter advertising the service.

Secondary objectives aim to: (1) assess the relative effectiveness of the two recruitment methods on biochemically validated 7-day point-prevalent abstinence rates at the 6-month follow-up; (2) compare the cost-effectiveness of the two invitation methods; (3) assess the relative effectiveness on prolonged abstinence measured by self-report of not smoking for periods of 7 days to 24 weeks at the 6-month follow-up; (4) assess the number of smokers attending the taster session and the number of smokers completing the 6-week NHS smoking cessation course; (5) assess the number of quit attempts made and any reduction in daily cigarette consumption; (6) determine predictors of attendance at the services, and of attendance at the taster sessions (in the Intervention group); (7) explore reasons for non-attendance and barriers to attendance at the NHS SSS; and (8) explore the effectiveness of the intervention by socioeconomic status, and social deprivation.

## Method

### Design and setting

The study is a randomised controlled trial (RCT) of a primary care population, utilising general practices to recruit smokers to the NHS SSS. Conducted in two stages, the pilot phase, in two areas served by a NHS SSS, aimed to ascertain rates of recruitment, the rate of uptake in attendance at the taster session, and to establish that the difference in uptake of smoking cessation services between the Intervention and Control groups is greater than zero, before proceeding to the full trial in a further eight areas that are representative of the English SSS.

The trial is conducted as a collaboration between University College London (UCL) and the Universities of Cambridge and York. It is being coordinated from UCL and is run through the UK Clinical Research Collaboration (UK CRC) registered UCL Clinical Trials Unit (PRIMENT). Ethical approval for the study was granted by the South West London Research Ethics Committee, and R & D approval will be obtained from all participating SSSs.

### Risks and benefits for trial participants

Ultimately this study aims to help participants to stop smoking, and risks for trial participants are minimal. Patients are informed in the patient information leaflet of the benefits of attending the NHS treatment services in order to obtain help to change their smoking behaviour to be of benefit to their health. It is unlikely that there will be any adverse effects for trial participants. However, anyone experiencing concern about their smoking habit as a result of the communication has the opportunity to attend the services to obtain help. Anyone experiencing any other kind of distress as a result of the assessment or intervention is referred to their general practitioner (GP) or practice counsellor.

All participants included in the trial will have given their written consent to take part, and for their data to be used in communications to them, and for any subsequent monitoring data obtained as a result of their use of the NHS services to be used for research purposes.

### Target population

The target group is smokers motivated to quit who have not recently attended the NHS SSSs.

#### Recruitment procedure

The Primary Care Research Network (PCRN) comprises eight clinician-led Local Research Networks, providing comprehensive geographical coverage of England. Team members work with GP practices to facilitate the involvement of staff and patients in clinical studies. We are working with these networks to identify practices in selected SSS areas, using Census data and Indices of Multiple Deprivation (IMD) (the Government’s official measure of multiple deprivation at small area level which provides a relative ranking of areas across England according to their level of deprivation) to select more practices in areas of high deprivation and of large ethnic communities to ensure full representation of smokers most in need of help, and to maximise the generalisability of the results.

Participating practices identify all smokers aged 16 years and over from their medical records. After screening by GPs to exclude anyone deemed to be unsuitable for the project (for example, severely or terminally ill) all remaining persons on the list are sent a brief screening questionnaire with a cover letter from their GP. A participant information leaflet describing the research is included, with a consent form to participate in the trial. Participants are also asked to provide consent for the release of relevant data from their attendance at the NHS services to the researchers, used to validate attendance and quit rates. The questionnaire data is used to assess the criteria for inclusion in the trial, and to provide information for the tailored letter. Patients have the option of returning the questionnaire to update their smoking status in their records only and not participating in the trial. A Freepost envelope is included for the return of the questionnaire to the practice, and non-responders are sent a reminder and duplicate questionnaire 3 weeks after the first mailing. All smokers returning the signed consent form and eligible to participate are randomised to the Intervention or Control group.

#### Inclusion/exclusion criteria

All current smokers willing to participate and returning the signed consent form, aged 16 years and over, able to read English, motivated to quit, and have not recently attended the NHS services are eligible for inclusion in the study. For the purposes of this research, motivation to quit is defined as answering ‘yes’ to either or both of the following questions: (1) are you seriously thinking of quitting in the next 6 months; and (2) would you think of quitting if appropriate help were offered at a convenient time and place. Exclusion criteria are minimal because the aim is to recruit all smokers into the services. However, smokers younger than 16 years old are excluded because of the need for parental consent to participate for this age group. Also excluded are any patients identified who are considered by the GP to be unsuitable for the project, for example, severely or terminally ill.

### Planned interventions

#### Control group

Participants are sent a standard generic letter from the GP practice advertising the local NHS SSS and asking the smoker to contact the service to make an appointment to see an advisor.

#### Intervention group

Participants receive the following:

(1) a brief motivational letter sent from the GP that includes information specific to the patient. The letter is personalised and tailored using known characteristics (age and gender), plus information obtained from the screening questionnaire (dependence, previous quit attempts). Information from medical records about the patient’s general health status and about chronic conditions (for example, heart disease, diabetes, lung disease) is also used to provide risk information and to offer help to improve their condition by quitting smoking. The amount of tailoring is maximised within the constraints of the short screening questionnaire and brief letter. The content of the letter has been developed in collaboration with GPs and primary care experts with knowledge of medical information available in records.

(2) an invitation and an appointment to attend a ‘Come and Try it’ taster session to find out more about the services. This taster session is run by advisors from the local SSS, and includes: an explanation that the advice and help offered by the NHS service is based on evidence, with a higher likelihood of success; information about the services offered, that is one-to-one or group sessions, the length of a session and the length of the course, nicotine replacement therapy (NRT) or other pharmacotherapy, as well as behavioural support; information about what to expect when they attend and the content of advice (for example, help dealing with weight gain, the correct use of NRT, depression, expected outcomes); the expectations of the service, that is willingness to set a quit date and a rule of not smoking a single puff after the quit date; a measurement of CO level with an interpretation; a 5-min video showing group and one-to-one sessions in progress, and testimonials from previous successful attendees, produced by UCL Media Services in collaboration with Camden SSS; the opportunity to ask questions about the service.

Each SSS runs eight to twelve taster sessions. Approximately 50 participants are invited to each session which lasts about 1 hour and at the end of the taster session attendees are encouraged to sign up to a group or one-to-one session at the SSS at a time convenient to them.

A standard protocol for the taster sessions was developed in consultation with one of the co-investigators (SG) and the NHS Centre for Smoking Cessation and Training (NSCTC) to conform to national guidelines [28]. Three to four advisors in each SSS, already trained to give smoking cessation advice in group and one-to-one sessions, are trained to lead the taster sessions according to this protocol, standardised on the understanding that, while services may differ in the way they are organised, the protocols for delivering advice are uniform. In order to achieve standardisation to the protocol, the training is manualised and includes an explanation and clarification of the study protocol and procedures. The same advisors will lead all sessions in each SSS. The taster sessions are audio-recorded, with the consent of the attendees to ensure fidelity to the protocol.

The intervention is further enhanced by the addition of a repeated personal letter with a further invitation sent 3 months after the original to all participants who fail to attend a taster session. This is consistent with recommendations made by Lichtenstein [7], who proposed that, with repeated advice over time, a greater proportion will be likely to respond.

The standard generic letter and an example of the personal risk letter and taster session invitation are included in the Additional Material files (see Additional file 1).

### Data management and randomisation

The patient-level data collected in this trial comprise information downloaded from practice records, and information provided by participants on the consent form, the baseline questionnaire, and in the follow-up telephone interviews.

The practice record information is used to: (1) generate letters inviting patients to participate in the trial, sent from the practice; and (2) generate the tailored and generic letters, also sent from the practice. Purpose-written programs written in Visual Basic for Applications (VBA) which read and write Excel and Word files are used to generate these letters.

#### Procedure for generating letters

Data from medical records are downloaded to an Excel spreadsheet and stored on a practice computer. The data are backed up on memory sticks, or on another practice computer. Using a computer program written in VBA, stored on a laptop, these data are used to create a file of invitation letters in MS Word. Potential participants who live in the same household are identified by the program to ensure that only one person from the same household receives a screening questionnaire. The file of invitation letters is copied to a practice computer and a back-up disk and deleted from the laptop. Each patient returning the completed questionnaire, and willing and eligible to participate, is coded as ‘participant’ on the Excel spreadsheet and the name and address information checked and updated if necessary on the spreadsheet against the consent form. Participants are exported to a new spreadsheet. The questionnaire data necessary to produce the intervention letter are coded into the new spreadsheet.

A second computer program, also written in VBA and stored on a laptop: (1) allocates to the participant a study ID number; (2) randomises participants to the Control or Intervention group; and (3) combines the data from the baseline questionnaire and medical records with the correct messages from a message library written using Microsoft Word, to generate two Word files, containing the tailored letters for intervention participants and the generic letters for control participants, respectively.

Patients who return the questionnaire with written consent, but who are not eligible to take part in the study are sent a letter thanking them for responding and informing them that they do not fit the study criteria, and patients who return questionnaires outside the timeframe for processing are sent a similar letter informing them that the recruitment period is ended. Both letters are sent from the GP practice and contain information about the local NHS Stop Smoking Service, advising the smoker to contact the service for more information or to speak to an advisor.

#### Security

This procedure is managed within each practice by practice and research staff. All data files and back-up media remain in the practice until all eligible participants have been randomised and the tailored and generic letters have been generated. At this point proprietary encryption software (Truecrypt) is used to create two separate encrypted files containing: (1) personal information (that is, name and address); and (2) medical and questionnaire information (including information on medical conditions from the patient records). These files are copied to a CD and taken to the study centre at UCL where they are copied and stored on separate encrypted volumes on a UCL server. This is the only point in the procedure at which electronic data leave the practice.

The consent forms and questionnaires are also taken from the practice to the study centre and stored in locked filing cabinets after the questionnaires have been scanned to enter the remaining data, also stored on encrypted volumes on a UCL server.

All data files are deleted from the laptop before leaving the practice. The only files kept on the laptop are the purpose written computer programs, the message library, the randomisation tables, the practice letterhead file, and the file containing GP signature.

The files from different practices will later be merged into a single file of personal information and a single file of medical and questionnaire information stored on separate encrypted volumes at UCL. A subset of this information will be used by research interviewers to conduct the follow-up telephone interviews. All electronic data will be stored on encrypted volumes on a UCL server.

This is a highly secure data management system that avoids the need for web-based transfer of electronic patient-level data.

#### Randomisation

Randomisation, at the level of the study participant, is embedded into the computer program according to an externally constructed randomisation plan, using permuted blocks. Participants are randomised in the ratio 3:2 (intervention:control) within practice, stratified by gender, and using a block size of 5. For each practice, a computer program is run to create two randomisation tables, one for men and one for women. Each table consists of 500 rows. In one column, there is a sequence of 2 s and 1 s in blocks of 5 (for example 1,1,2,2,1). This sequence is created by listing all possible permutations of three 1 s and two 2 s (10 in all), then repeatedly selecting one permutation at random (with replacement) and adding each selection to the sequence. This procedure uses the random number generating function rnd in Microsoft VBA. For each table, the Randomize statement is used to initialize the random number generator with a seed based on the system timer. Having created the tables for a given practice, another computer program is used to allocate participants from that practice to condition by selecting the first unused code (1 or 2) from the table for men or the table for women, depending on the participant’s gender, and then marking that code as used. If the information about gender is missing for a participant, the randomisation table to be used is selected at random. Any imbalances will be controlled for in the statistical analysis using covariates that are identified prior to examining the trial data. The use of a computer program that enforces randomisation after consent and baseline data entry ensures that concealment is preserved and differential entry prevented.

#### Methods to protect against other sources of bias

By randomising at the level of participant rather than by practice, there is a slight risk of contamination by communication between patients at the same practice allocated to different conditions. While we consider this risk to be low, the following measures reduce the risk further: identify potential participants who live in the same household and ensure that only one person from the same household receives a screening questionnaire; monitor attendance at the taster sessions, to ensure that anyone attending who has not received an invitation is recorded and checked against participants in the Control group; measure the amount of contamination at follow-up by: (i) asking participants whether they have attended a taster session, and if not, whether they personally know or have spoken to anyone else who has been invited to the NHS stop smoking services; and (ii) validation of self-report by keeping a record of attendance at the taster sessions.

It is not possible to blind participants to the receipt of a personally tailored letter, and invitation to a taster session. While the personal letter is generated in the practice by Research Assistants, the remainder of the research team in all cases are blind to the allocation of the participant, which will be enforced by the data management. In follow-up interviews, the interviewer will be blinded to the allocation of the respondent in order to avoid bias in outcome assessment.

External validity of the sample is estimated by comparing data on non-responders to the invitation to participate with that of responders. Data are anonymised by removing names, addresses, and NHS numbers of non-responders from the database. Gender, date of birth (converted to age), and postcode (converted to an IMD score via GeoConvert) are used to calculate means within each practice and compare to those of responders.

#### Duration of treatment period and follow-up

**Figure 1:**
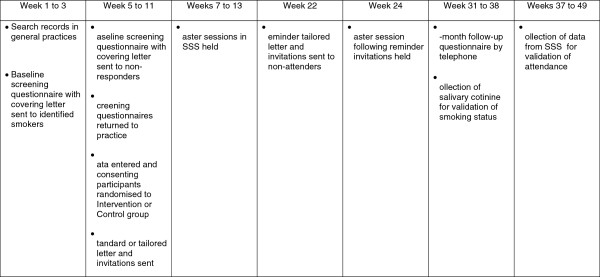
Timing of assessments, intervention, and follow-up.

The process from the searching of records to identify smokers to the completion of the follow-up takes approximately 38 weeks. Recruitment takes place over 7 weeks, with three practice visits by members of the research team to process responses. Four taster sessions are held in each area, three to coincide with the visits to process responses, and a final one to which all remaining non-attenders are invited. Figure 1 shows detail of the timing of assessments, intervention, and follow-up.

### Measures

#### Baseline measures

Lichtenstein [7] found that readiness, motivation, and dependence were positively related to follow through, readiness defined as smokers planning to quit in the next 6 months. The baseline screening questionnaire therefore uses the definitions and measures of motivation and readiness used by Lichtenstein [7] in order to provide the right encouragement to those who might be prompted to quit. In addition to screening for inclusion criteria, also assessed are demographics, dependence on nicotine (number of cigarettes per day and time from waking to first cigarette), smoking history (age started and previous quit attempts), and determination to quit.

#### Outcome measures

##### Primary

The proportion of people entering the smoking cessation service (that is, attending the first session of a 6-week course) over a period of 6 months from the receipt of the invitation letter. Self-reported attendance data will be validated by records of attendance at the NHS SSSs.

##### Secondary

Secondary outcome measures are: (1) 7-day point prevalent abstinence at the 6-month follow-up, validated by salivary cotinine for all participants reporting abstinence in both the Intervention and Control groups; (2) prolonged periods of abstinence of 7 days to 24 weeks measured by self-report; (3) self-reported changes in daily cigarette consumption, quit attempts, and changes in motivation and intention to quit in continuing smokers; (4) use of NRT or Zyban or Champix and other smoking cessation aids; and (5) the number completing the 6-week NHS course.

##### Process measures

Process measures are: (1) the number of smokers attending the taster session (Intervention group only); (2) perception of the taster session; (3) perception of the personal invitation letters; and (4) reasons for non-attendance at the taster session and barriers to attendance at the NHS services.

##### Health economic measures

The economic component will estimate the cost of providing the interventions, using primary cost data from an NHS and personal social services (PSS) perspective as recommended by National Institute for Health and Clinical Excellence (NICE) guidance [29]. We will also measure patients’ use of health and social care services using comprehensive service use questionnaires as employed on a number of other trials in the addiction field. Quality-adjusted life years (QALYs) will be calculated from the EQ-5D questionnaire [30] and combined with cost data in the cost-utility analysis.

### Evaluation procedure

Research interviewers, independent from the service providers, will conduct follow-up interviews 6 months after the date of randomisation, by telephone, to assess attendance at the services, current smoking status, daily cigarette consumption, reasons for non-attendance, and barriers to attendance in all participants. Interviewers will make a maximum of 10 attempts to contact the participant at varying times of day and on different days before classifying the participant as lost to follow-up.

All self-reported attendance at the NHS SSS will be validated by records of attendance. All participants will be asked if they attended a taster session; those answering negatively will be asked if they know or have spoken to anyone who attended a taster session. Participants claiming 7-day abstinence will be asked to provide a salivary cotinine sample to bio-chemically validate 7-day point prevalent smoking cessation at a 6-month follow-up [31]. Samples will be obtained by post using a saliva sample kit. As the cotinine content can be affected by continued use of NRT, usage at the time the sample is given will be assessed by questionnaire. A £5 Marks and Spencer voucher will be included with each kit, and a further £5 voucher sent upon return of the kit to maximise kit return [32].

Attendance at the NHS SSS could be taken up at any time during the 6 months between receipt of the invitation (Intervention Group) or standard letter (Control Group) and the 6-month follow-up. Hence, at 6 months some participants could be at varying stages of completion of a course, and at varying stages of a quit attempt. Therefore secondary outcome measures include self-reported prolonged abstinence of periods of up to 24 weeks, a measure that can also apply to those not attending the NHS service.

We will also assess 4-week abstinence in those attending the SSSs using the NHS monitoring data collected by smoking cessation advisors. These monitoring records can be used to compare quit rates of clients proactively recruited through our intervention with those of other attendees at the NHS SSSs.

We will measure perception of the personal invitation letters and of the taster session using measures from our previous trials of tailored feedback adapted to apply to the personal invitation letters [21,33]. Reasons for non-attendance at the taster session and barriers to attendance at the NHS services will be assessed using open rather than closed questions to ensure that respondents are not inhibited in their answers to those which the researchers consider to be relevant. All process measures will be included in the telephone follow-up interview 6-months after the date of randomisation. Perception of the taster session will also be assessed by an evaluation form immediately after each session.

### Sample size and power calculations

Recent evidence from the study of Murray and colleagues suggests that attendance at NHS services can be increased by 7.7% (from 8.9% to 16.6%) using a proactive intervention [18]. To detect an effect of this size at 90% power and alpha of 0.05 would require a sample of 420 participants per group. However, in the absence of other similar trials, we might assume that the uptake of services in those who receive the tailored letter and the taster session could be lower than that reported by Murray. Hence, assuming an estimated increase of 4.6% (from 8.9% to 13.5%, OR 1.65) we would require 1,029 participants per group, 2,058 in all, to detect this difference as statistically significant at the 5% level with 90% power.

Practices will be recruited from 10 different NHS SSSs. The taster sessions in each SSS will be run by the same four advisors comprising 10 therapist clusters. Thus before adjusting for clustering we would expect 103 patients per cluster. While the intervention is manualised and structured training run to reduce the variability between the interventions delivered in each SSS, to account for any persistent therapist effects that will be applied to those randomised to receive a taster session, assuming a therapist intraclass correlation coefficient (ICC) of 0.005 (in the absence of any published data), and a therapist cluster size of 103, requires further inflation of our existing sample size by a factor of 1.51 only in the Intervention group where the effects will occur. Thus, 1,554 will receive the tailored letter and taster session, 2,583 participants in total.

The same RCT [18] found validated quit rates at 6 months of 4% *versus* 2.2% (a difference of 1.8%) in the Intervention and Control Groups. With 2,583 participants (distributed between Intervention and Control groups as described above), we will be able to demonstrate a slightly larger difference of 2.2% with 80% power.

#### Planned recruitment rate

Practices generally identify 13% to 22% of their patients as smokers [34], depending on the characteristics of the patient population, and the accuracy and completeness of the records. Therefore six practices in each of 10 SSSs with a list size of > 4,000 would give approximately 240,000 patients and, assuming a conservative smoking prevalence of 15% in patients aged 16 years and over, 36,000 smokers. Based on previous studies [18,26] we estimate a response rate of 7% from smokers motivated to quit, from two mailings. This will secure 2,520 participants, meeting the requirements of the sample size calculation.

Initial recruitment to the pilot phase of the RCT was planned in 12 practices in two SSSs. Using the assumptions above this would have secured 504 participants. We then planned to proceed to the full RCT to recruit a further 2,016 participants in a further eight SSSs after conducting a first stage analyses to confirm that: (1) a 7% response rate from participants giving consent and agreeing to randomization has been achieved; and (2) a preliminary analysis suggesting a difference in uptake of smoking cessation services between the Intervention and Control groups greater than zero.

Based on previous studies using follow-up by telephone [21], approximately 20% to 25% attrition is expected.

### Statistical analysis

#### Pilot phase

Recruitment during the pilot phase was estimated to be approximately 20% of the total sample. The methods used in the pilot phase are essentially the same as those used in the full trial to enable combination of the data from both phases for analysis. However, lessons learnt on recruitment strategies from the pilot phase will be applied to the main trial.

#### Main analysis

Baseline characteristics of participants will be summarized. Chi-squared tests will compare binary outcomes between the Intervention and Control Groups (for example, attendance at the services, point prevalent abstinence), with logistic regression to take into account any imbalance in important baseline characteristics between the groups; these factors will be nominated prior to examination of the trial data. Continuous variables (for example, reduction in daily cigarette consumption) will be compared with the two-sample *t*-test, and with multiple linear regression to account for important characteristics. Odds ratios for differences in means or medians (as appropriate) will be quoted together with their 95% confidence intervals. Loss to follow-up after randomisation will be reported. Analyses will be based on intention-to-treat (that is, those lost to follow-up will be assumed to be still smoking) with sensitivity analysis to examine the influence of loss to follow-up.

Specifically we will conduct an analysis on multiple imputed missing outcome data at 6 months. The imputation model will use observed baseline covariates and outcome data. Estimates of the attendance at NHS smoking cessations SSS at 6 months will be compared between the Intervention and Control groups. A sensitivity analysis will then be possible based on our worst case imputation (that is, our assumption of non-attendance for all those lost to follow-up) [35,36]. A logistic regression model will be used to adjust for baseline sociodemographic variables. The general practice will be included in the model as a random effect. In addition to the intention-to-treat analysis we shall estimate the causal effect of the intervention using CACE, the complier average causal effect estimator (or equivalently, an instrumental variables estimator) [37]. In the CACE analysis we will consider attendance at the first NHS smoking cessation service in terms of a binary variable as well as a dose effect (that is, the number of sessions attended in total). We shall take a parallel approach to the analysis of secondary outcomes (for example, smoking cessation).

#### Planned subgroup analyses

We will examine the predictors of attendance in the two groups (that is, in those proactively recruited and those self-referred after standard advertising). We will also explore any delayed effect of sending repeat reminders to smokers on the uptake of service, and any differences in attendance due to seasonal variations. We are aware, however, that the study will be inadequately powered to do any detailed subgroup analyses on specific groups of smoker such as those of lower socioeconomic status and hence we will merely explore for trends.

We will also estimate if the quit rate in the recruited sample is similar to that of services based on figures from previous quarterly returns.

#### Economic analysis

The economic analysis will measure and value the costs of delivering the interventions and the wider changes in health and social care costs. Intervention costs will be calculated by computing the costs of the programmes, which will include materials used in the programmes and the time spent by health professionals in service delivery. These costs are then attributed to patients receiving the interventions. Patients’ wider utilisation of health and personal social services resources in the preceding period are also recorded at baseline and follow-up. The total costs of the health and personal social services resources are calculated using national unit costs from a range of published sources.

The total costs to health and personal social services are calculated, as recommended by NICE guidance [29], and combined with outcome data to generate the estimated cost per QALY. QALYs are derived from the EQ-5D questionnaire [30] administered at baseline and follow-ups. The probability of the tailored letter being cost-effective over and above the generic letter at NICE QALY threshold values will be explored using cost-effectiveness acceptability curves [38].

#### Proposed frequency of analyses

An analysis will be conducted on completion of the follow-up to the pilot study. Following this there will be no further analyses until full recruitment and follow-up has been achieved.

#### Service user involvement

The trial is embedded in the NHS through the inclusion of a Stop Smoking Service Manager as a co-applicant, who is involved in the design, conduct, and analysis. In addition, a suitable past successful user of the service in Camden has been invited onto the Trial Management Group and is involved in the study from the design stage onwards. Thus, the interests of all parties and the views of the public are fully represented in the conduct of the study.

## Discussion

Personal risk information, generated by computer, is a simple and inexpensive intervention which, if the trial demonstrated benefit, could be widely replicated and delivered cost-effectively to a large proportion of the smoking population, prompting more quit attempts, and increasing referrals to NHS specialised smoking cessation services. The programme could be made available to practices, these letters, tailored to the requirements of each individual, offering GPs and practice nurses an efficient way of integrating referrals to the smoking cessation service into a busy primary care practice. Moreover, the introduction of a taster session delivered by existing NHS smoking cessation advisers could be easily implemented into the practice at a small additional cost. A modest success rate could have a large effect on uptake of services given its recruitment potential, and make a valuable contribution to public health by lowering smoking prevalence.

## Trial status

The pilot phase of the trial has been completed. Recruitment was carried out between February and April 2011, in Camden and Oxfordshire, both areas with high proportions of ethnic minorities, and the follow-up was completed between September and December 2011.

The aims of the pilot phase were to assess the feasibility of the procedure (that is, searching medical records and mailing screening questionnaires, the generation of the tailored letters, the randomisation and delivery of the intervention), to ascertain recruitment rates and to assess the uptake of the taster sessions, and to establish that the uptake of smoking cessation services in the Intervention group was greater than in the Control group (that is, the difference in proportions, intervention minus control, is greater than zero).

Successful achievement of these aims has allowed us to proceed to the full trial. Recruitment slightly below the original estimate led to a revision of the planned recruitment rate from 7% to 5.5%, and to a revision of the number of smokers identified to receive the initial mailing, in order to meet the target sample size (Figure 1).

### Changes due to additional funding

As a result of the successful recruitment on the study, additional funding was approved in July 2012 to carry out additional work which will build on the study and maximise its utility. The following changes have been implemented:

(1) In addition to the original primary outcome (the proportion of people entering the smoking cessation service over a period of 6 months), 7-day point prevalent abstinence at the 6-month follow-up, validated by salivary cotinine is now also included as a co-primary outcome. This will allow us to assess whether this intervention also translates to increased quit rates, and if the quit rates in people attending as a result of this intervention differ from the usual quit rates in NHS services. This change necessitated a recalculation of the sample size needed. Assuming quit rates of 4% *versus* 2.2% in the Intervention and Control groups (mimicking the findings of Murray *et al.*[18]) an 80% increase in the sample size is required, to 1,793 in the Control group and 2,707 in the Intervention group (assuming the same therapist effect as the original protocol), a total of 4,500. A sample of this size gives 85.4% power to detect a difference of 1.8% at the 5% significance level. The same sample size would have 95% power to detect the difference between quit rates of 4.4 and 2.2% (doubling of the quit rate). Based on current recruitment figures, we estimate that an additional eight SSSs (48 GP practices) will recruit 2,060 participants, giving a total of 4,580 and meeting the requirement of the power calculation.

(2) In the pilot phase of the study we assessed barriers to attendance at the NHS services using an open question. For the remainder of the study we will use the Treatment Barriers Questionnaire, a 40-item measure of reasons for not entering smoking cessation programs that has been recently validated on a low socioeconomic population in the USA [39]. This questionnaire will allow us to assess different aspects of smokers’ decisions to attend a group or therapy session and highlight any misconceptions or lack of awareness of the service offered. It will also allow us to explore associations with demographic and dependence factors, as well as validating the questionnaire on a UK population. The Treatment Barriers questionnaire will be mailed to approximately 3,500 participants who report not attending the SSS and who agree to complete an additional questionnaire.

(3) Taster sessions are being recorded to ensure fidelity to the protocol. Assessing this fidelity can help to address factors that might have impacted on subsequent attendance and quit rates. Full analysis of the recording of the Taster sessions, using thematic analysis, will be carried out to allow the exploration of differences in style and delivery of the intervention and their impact on subsequent attendance and quit rates.

Recruitment to the main trial is now under way in a further 16 areas that are representative of the English SSS. The target of 4,500 participants is expected to be reached by October 2013.

**Figure 2:**
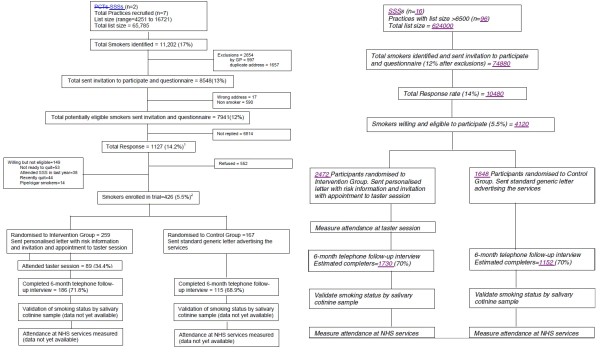
Consort diagram of the flow of participants through the trial.

The flow of participants in the pilot phase and the planned flow of participants through the main trial are summarised in Figure 2.

## Abbreviations

CACE: Complier average causal effect estimator; CO: Expired-air carbon monoxide; GP: General practitioner; ICC: Intraclass correlation coefficient; IMD: Index of multiple deprivation; NHS: National Health Service; NICE: National Institute for Health and Clinical Excellence; NRT: Nicotine replacement therapy; PCRN: Primary Care Research Network; PCT: Primary Care Trust; PSS: Personal social services; QALY: Quality-adjusted life year; RCT: Randomised controlled trial; SSS: Stop Smoking Service; UCL: University College London; UK CRC: United Kingdom Clinical Research Collaboration; VBA: Visual Basic for Applications.

## Competing interests

The authors declare they have no competing interests.

## Authors’ contributions

HG is the PI. The study was conceived and designed by HG, IN, and SS. HG, IN, and SS contributed to writing the protocol, and the development of the tailored intervention letters. RM is the trial statistician and advised on matters relating to power and sample size calculations and randomisation. SP is a health economist and commented on the protocol and contributed to matters relating to economic issues. SG, a Stop Smoking Service Manager, developed the taster sessions and advisor training protocol, and advised on service delivery aspects of the study. All authors read and approved the final manuscript.

## Supplementary Material

Additional file 1 **Generic standard letter. **Example of the personal risk letter and taster session invitation. Click here for file
