# Supplementary material for: Evaluating the effectiveness of using personal tailored risk information and taster sessions to increase the uptake of smoking cessation services: study protocol for a randomised controlled trial
Source: Trials. 2012 Oct 18;13:195. doi: 10.1186/1745-6215-13-195 (PMC3563550; doi:10.1186/1745-6215-13-195)
Supplement: Additional file 1 — Generic standard letter. Example of the personal risk letter and taster session invitation. [file 1745-6215-13-195-S1.docx]

**Generic control letter**

***PRACTICE LETTERHEAD***

Dear

You are being sent this letter as part of your participation in the Start 2quit project.

I recommend that you consider stopping smoking, and invite you to contact the Stop Smoking Services to arrange to see an advisor.

The service is available free to offer information, advice and support to all smokers interested in giving up, and also offers advice on the use of medications such as Nicotine Replacement Therapy and Champix.

You can arrange to join a group, or get individual advice in a one-to-one session, to help you stop smoking and stay stopped. For more information or to speak to a specialist advisor please call the ________Stop Smoking Service on ________.

Yours sincerely

GP signature

**Personal tailored risk intervention letter**

***PRACTICE LETTERHEAD***

**Personal Health Risk Report and Taster Session Invitation**

Dear Mrs Smith

You recently filled in a questionnaire for the **Start2quit** project. This letter is based on your answers in the questionnaire and on your medical records. It is written for you personally and gives you advice about smoking. We are also inviting you to a Taster Session to help you to become smokefree and improve your health.

**Your personal risk**

Based on your smoking habits and your personal health, your current risk of developing a further serious illness and suffering an early death is very high compared to a non-smoker or ex-smoker of your age.

Your records show that you also have diabetes, and even by smoking only 10 cigarettes a day you are seriously increasing your risk of high blood pressure leading to heart attack and kidney disease. By going smokefree now, you can prevent further decline and begin to heal.

**Take control and change your life**

Stopping smoking is the single most important thing that you can do to improve your health and quality of life. The good news is that if you quit now, at 37, you have greater chance of preventing any further complications and can halve your additional risk of contracting other diseases. By stopping smoking you will slow the progress of your existing condition and live with better health for longer. We recommend that you consider quitting without delay. **It could be the best thing you will ever do for yourself.**

**Don’t do it alone**

You might think it is hard to stop but you don’t have to do it alone. Help and support is available. The NHS Stop Smoking Service offers free personal support to help you. You have previously quit for a few days. Joining a stop smoking group or getting one-to-one support will increase your chances of staying quit and becoming smokefree. You will also feel less alone and gain the support of other people who are quitting.

**A place is reserved for you**

So that you can find out more about the Stop Smoking Service, we are inviting you to a ‘Come and Try it’ session at Camden Town Hall on Wednesday 10^th^ January 2011 at 7pm. Please bring the Invitation Card enclosed with you. If you cannot attend this session, please contact Leanne Gardner on 020 7794 0500 extn:36719. We can offer you an alternative time or an immediate appointment with an advisor.

With very best wishes

GP signature

**Taster session invitation**

**Susan Smith**

Invitation to a

**‘Come and Try it’**

**Stop Smoking Session**

at <*place*>

on <*day> <date>* at *<time>*.

*Please bring this card with you to the session.*

If you are unable to attend, please contact <*name*> on <*tel number*>
